# LDL/HDL cholesterol ratio is associated with new-onset NAFLD in Chinese non-obese people with normal lipids: a 5-year longitudinal cohort study

**DOI:** 10.1186/s12944-021-01457-1

**Published:** 2021-03-25

**Authors:** Yang Zou, Ling Zhong, Chong Hu, Mingchun Zhong, Nan Peng, Guotai Sheng

**Affiliations:** 1 Department of Cardiology, Jiangxi Provincial People’s Hospital, Aiguo 152 Rd, Nanchang, 330006 China; 2 Department of Pediatrics, Lishui People’s Hospital, No. 15 Dazhong St, Lishui, 323000 China; 3 Department of Gastroenterology, Jiangxi Provincial People’s Hospital, Aiguo 152 Rd, Nanchang, 330006 China

**Keywords:** Non-obese, Longitudinal study, Lipoprotein ratios, LDL/HDL cholesterol ratio, Nonalcoholic fatty liver disease

## Abstract

**Background:**

Low-density lipoprotein to high density lipoprotein (LDL/HDL) cholesterol ratio has been reported to predict the risk of many metabolic diseases. However, the association between the LDL/HDL cholesterol ratio and nonalcoholic fatty liver disease (NAFLD) has not been established.

**Methods:**

A longitudinal cohort design was adopted in this study; 9767 non-obese subjects without NAFLD were included and analyzed. The subjects were grouped according to the quintile of LDL/HDL cholesterol ratio. The cumulative incidence of NAFLD and the independent effect of the LDL/HDL cholesterol ratio on NAFLD during 5 years of follow-up were calculated using the Kaplan-Meier method and Cox proportional-hazards regression model.

**Results:**

During the 5-year follow-up period, 841 subjects were diagnosed with new-onset NAFLD, and the 1-, 2-, 3-, 4-, and 5-year cumulative incidence rates of NAFLD were 1.16, 4.65, 8.33, 12.43, and 25.14%, respectively. In the multivariable-adjusted Cox proportional-hazards regression model, the LDL/HDL cholesterol ratio was significantly associated with the risk for NAFLD (HR: 1.66, 95% CI: 1.38–1.99, *P* trend< 0.001), especially among young people (HR: 3.96, 95% CI: 1.50–10.46, *P* interaction< 0.05). Additionally, receiver operating characteristic curve analysis showed that the LDL/HDL cholesterol ratio was better than HDL cholesterol and LDL cholesterol in predicting new-onset NAFLD.

**Conclusions:**

LDL/HDL cholesterol ratio is an independent predictor of NAFLD in Chinese non-obese people with normal lipids, and its predictive value is higher than that of other lipoproteins. In clinical practice, the LDL/HDL cholesterol ratio can be used to identify people at high risk of NAFLD.

**Supplementary Information:**

The online version contains supplementary material available at 10.1186/s12944-021-01457-1.

## Background

Nonalcoholic fatty liver disease (NAFLD) is the most common liver disease worldwide and the main cause of various liver diseases [1–3]. It is also a risk factor for a series of metabolism-related diseases, such as metabolic syndrome, diabetes, and cardio-cerebrovascular disease [3–5]. It has become an important issue of global public health in the twenty-first century [3]. As reported, the global prevalence of NAFLD is about 25% in adults [6, 7], while in Asia, nearly 30% of adults are affected by NAFLD, and this number is still increasing with the prevalence of obesity [6, 8]. The potential huge disease burden of NAFLD will place great pressure on the security of the medical system and socioeconomic system [3, 6, 9]. At present, there is no effective drug approved by regulatory authorities for the treatment of NAFLD. The prevention and treatment of NAFLD are mainly through lifestyle improvement and weight loss [1, 10]. Therefore, the prevention strategy for identifying and adjusting the potential risk factors is ideal for reducing the risk of NAFLD in the population.

It is well known that obesity is closely associated with the prevalence and severity of NAFLD [3, 6, 11]. However, in recent years, many studies have pointed out that non-obese people are also prone to NAFLD [12, 13]. In previous reports, the prevalence of NAFLD among non-obese people in China was 8.61% [14], and similar results (7.4%) were reported in the United States [15]. Additionally, several recent large cohort studies have shown that compared to obese NAFLD patients, non-obese NAFLD patients are at significantly increased risk for metabolic syndrome and hypertension [16, 17], making early detection and intervention of risk factors in non-obese NAFLD patients extremely important.

Lipid metabolism is also closely related to NAFLD. Previous studies have shown that higher HDL cholesterol is an independent protective factor for NAFLD, while triglycerides (TG), LDL cholesterol, and total cholesterol (TC) are associated with an increased risk of NAFLD [1, 18]. These lipoproteins have been recommended for screening people with a high risk of NAFLD in clinical practice [18]. Recently, some studies have pointed out that the LDL/HDL cholesterol ratio can simultaneously evaluate LDL cholesterol and HDL cholesterol, and its performance in predicting the risk of cardiovascular and cerebrovascular metabolic-related diseases is better than that of a single lipoprotein [19–21]. At present, there are no research data on the association between NAFLD and the LDL/HDL cholesterol ratio. Considering the prevalence of non-obese NAFLD, which has not received widespread attention, the relevant data are still immature. Therefore, this study is based on a large sample of longitudinal cohorts to evaluate the association between the LDL/HDL cholesterol ratio and the risk of new-onset NAFLD in non-obese people with normal lipids.

## Methods

### Study design and subjects

This research conducted a post hoc analysis of a longitudinal cohort from Wenzhou People’s Hospital [22]. The data were from the Dryad database, which was uploaded and shared by Sun et al. [23]. The longitudinal cohort recruited 33,153 subjects who underwent physical examination at Wenzhou People’s Hospital from the beginning of 2010 to the end of 2014. According to the purpose of the secondary analysis, this study excluded subjects who met the following criteria at baseline: i) subjects who were still taking oral antihypertensive, hypoglycemic and lipid-lowering drugs at baseline; ii) subjects with liver disease; iii) subjects who lacked follow-up data; iv) subjects with BMI ≥ 25 kg/m^2^ [24]; v) subjects with excessive alcohol consumption (weekly alcohol consumption: male> 140 g, female> 70 g); and vi) subjects with dyslipidemia (fasting sample: HDL cholesterol < 1.04 mmol/l, TG > 1.7 mmol/l, LDL cholesterol > 3.12 mmol/l and TC > 5.2 mmol/l). Finally, this study analyzed 9767 subjects who met the criteria. Because of the previous study has been approved by the institutional review boards of Wenzhou people’s Hospital and the written informed consent of the participants [22], there was no need to apply for ethical approval again for this study.

### Data collection and follow-up

Use standardized self-filling spreadsheets to obtain detailed information on basic clinical data, including height, age, weight, gender, and diastolic and systolic blood pressure (D/SBP). Venous blood samples were taken by experienced medical staff after overnight fasting and analyzed by an AbbottAxSYM analyzer. The laboratory parameters included in this study were DBIL: direct bilirubin; ALP: alkaline phosphatase; Cr: creatinine; TP: total protein; FPG: fasting plasma glucose; UA: uric acid; TC; ALB: albumin; TG; AST: aspartate aminotransferase; GLB: globulin; TB: total bilirubin; HDL cholesterol; BUN: blood urea nitrogen; ALT: alanine aminotransferase; LDL cholesterol; and GGT: gamma glutamyl transferase.

The beginning of the follow-up was from the clinician’s first NAFLD evaluation to the subjects, and then the NAFLD evaluation was performed annually by abdominal color ultrasound, while other baseline changes and medication status were not recorded. The follow-up period was 5 years.

### Diagnosis of NAFLD

Diagnosis of NAFLD was performed using the abdominal color ultrasound diagnostic procedure published by the Chinese Society of Liver Disease [25], and the main contents included the following five items: i) diffuse hyperechoic liver (compared to spleen and kidney); ii) enlarged liver with blunt, rounded edges; iii) visibility of detailed structures in the liver is reduced; iv) the envelope of the right liver lobe and diaphragm with unclear or nonintact display; and v) hepatic blood flow signal weakening. The diagnosis of NAFLD met the first of the above five items and any other items.

### Missing data processing

Missing data are a problem in almost every observational study, while in the data set analyzed in this study, missing data accounted for 8.85% of all variables (Additional file 1, Supplementary Table 1). To reduce the bias caused by missing covariables, which cannot reflect the statistical efficiency of the target sample in the modeling process, the missing data in this study were imputed using multiple imputation [26], and five imputations were created. In this study, all the analysis steps were calculated and compared in the original dataset and imputed dataset, and the results showed that the original data were consistent with the core results of the imputed data. Therefore, the results of multiple regression analysis in this study adopted the values of imputed data, in which the estimates from each imputation were combined according to Rubin’s rules [27]. Additional file 1 provides a detailed statistical analysis process of the original data and estimated data.

### Statistical analysis

The relationship between the LDL/HDL cholesterol ratio and NAFLD was evaluated with the LDL/HDL cholesterol ratio as a categorical variable (divided into five groups according to quintiles) and continuous variable; Kaplan-Meier method was used to calculate the cumulative incidence of NAFLD over time. The hazard ratios (HR) and 95% confidence intervals (CI) of the LDL/HDL cholesterol ratio to NAFLD risk were calculated by constructing the Cox proportional hazards regression model. To reflect the direction and size of the association between the ratio of LDL/HDL cholesterol and NAFLD between different models [28], five models were used, in which the crude model being unadjusted. Adjusted model I: only the most basic demographic variable; whereas model II was adjusted for variables that contributed more than 10% to the risk of LDL/HDL cholesterol ratio matching with NAFLD [29]. Adjusted model III was model II plus the variables with *P* < 0.1 in the univariate analysis, and adjusted model IV comprised all the noncollinear variables (Additional file 1: Supplementary Table 2). Simultaneously, the same steps were used as above to explore some subsidiary analysis of the relationship between HDL cholesterol, LDL cholesterol and NAFLD and to construct a receiver operating characteristic curve (ROC) to estimate the ability of the HDL cholesterol, LDL cholesterol and LDL/HDL cholesterol ratio to predict new-onset NAFLD. Additionally, to further verify the reliability of the positive relationship between the LDL/HDL cholesterol ratio as a continuous variable and new-onset NAFLD, the researchers continue to visualize the association between them through a generalized additive model (GAM, cubic spline smoothing) [30]. Finally, considering that the association between the ratio of LDL/HDL cholesterol and NAFLD may vary in some populations, the researchers conducted exploratory stratified analysis (gender, age and FPG) in some subgroups through the Cox model and used the likelihood ratio test to check the hierarchical differences to determine whether there was interaction.

All analyses in this study were carried out using Empower Stats Software (version 2.2) and R language (version 3.4.3). Judging the distribution of continuous variables by a quantile-quantile plot, the baseline variables were reported as the medians (interquartile ranges), frequencies (percentages) or means (standard deviations). The differences between groups were compared by the Kruskal-Wallis H test or chi-square test or one-way analysis of variance, and the linear trend between groups was tested by linear regression or logistic regression. *P* < 0.05 (two-tailed) indicates statistical significance.

## Results

### Subject characteristics

In this study, 9767 non-obese subjects with normal lipids were analyzed. The study population’s median age was 39 (31–51) years old; males accounted for 51.42% and females accounted for 48.58%. During the 5-year follow-up period, 841 subjects were diagnosed with new-onset NAFLD, and the 1-, 2-, 3-, 4-, and 5-year cumulative incidence rates of NAFLD were 1.16, 4.65, 8.33, 12.43, and 25.14%, respectively.

Table 1 summarizes the basic characteristics of the subjects grouped according to the quintiles by the ratio of LDL/HDL cholesterol as a continuous variable. In the group with a higher LDL/HDL cholesterol ratio, the NAFLD prevalence rate and LDL cholesterol level were higher, and the HDL cholesterol level was lower. Additionally, in the five groups from quintile 1 to quintile 5, it can be observed that most of the clinical baseline indexes increased gradually with the increase in the LDL/HDL cholesterol ratio; in contrast, HDL cholesterol showed a gradually decreasing trend (*P* trend< 0.001).

**Table 1:** Baseline Characteristics of participants

LDL/HDL cholesterol ratio	Q1 (≥0.2, ≤1.1)	Q2 (> 1.1, ≤1.3)	Q3 (> 1.3, ≤1.5)	Q4 (> 1.5, ≤1.8)	Q5 (> 1.8, ≤3.0)	*P*-value	*P*-trend
N (%)	1953	1952	1955	1952	1955		
Age, years	38.00 (31.00–50.00)	39.50 (31.00–50.00)	38.00 (31.00–51.00)	40.00 (32.00–51.00)	40.00 (31.00–50.00)	0.443	0.214
Gender						0.003	0.0004
Female (%)	978 (50.08%)	993 (50.87%)	950 (48.59%)	945 (48.41%)	879 (44.96%)		
Male (%)	975 (49.92%)	959 (49.13%)	1005 (51.41%)	1007 (51.59%)	1076 (55.04%)		
NAFLD (%)	58 (2.97%)	113 (5.79%)	145 (7.42%)	188 (9.63%)	337 (17.24%)	< 0.001	< 0.001
BMI, kg/m^2^	20.27 (18.95–21.72)	20.61 (19.26–22.19)	20.91 (19.47–22.37)	21.30 (19.84–22.83)	21.96 (20.44–23.38)	< 0.001	< 0.001
Weight, kg	53.95 (7.35)	55.22 (7.77)	56.61 (8.12)	57.93 (8.33)	60.38 (8.34)	< 0.001	< 0.001
Height, m	1.63 (0.07)	1.63 (0.07)	1.64 (0.08)	1.65 (0.08)	1.66 (0.08)	< 0.001	< 0.001
ALP, U/L	62.00 (52.00–76.00)	65.00 (54.00–79.00)	66.00 (56.00–81.00)	69.00 (57.00–81.00)	72.00 (59.00–86.00)	< 0.001	< 0.001
GGT, U/L	18.00 (14.00–24.00)	18.00 (15.00–24.00)	19.00 (15.00–26.00)	20.00 (16.00–27.00)	22.00 (17.00–30.00)	< 0.001	< 0.001
ALT, U/L	14.00 (11.00–20.00)	14.00 (11.00–19.00)	15.00 (11.00–21.00)	16.00 (12.00–21.00)	17.00 (13.00–24.00)	< 0.001	< 0.001
AST, U/L	20.00 (17.50–24.00)	20.00 (18.00–24.00)	20.00 (18.00–24.00)	21.00 (18.00–24.00)	21.00 (18.00–25.00)	< 0.001	0.942
TP, g/L	73.48 (4.21)	73.46 (4.06)	73.70 (4.14)	73.90 (3.96)	74.15 (4.07)	< 0.001	< 0.001
ALB, g/L	44.25 (2.87)	44.34 (2.75)	44.29 (2.69)	44.31 (2.61)	44.28 (2.77)	0.879	0.883
GLB, g/L	29.24 (3.99)	29.12 (3.78)	29.41 (3.83)	29.59 (3.75)	29.87 (3.93)	< 0.001	< 0.001
TB, μmol/l	11.30 (8.80–14.60)	11.10 (8.70–14.10)	11.20 (8.80–14.30)	11.30 (8.93–14.50)	11.50 (9.00–14.50)	0.422	0.579
DBIL, μmol/l	2.20 (1.70–2.90)	2.10 (1.60–2.80)	2.10 (1.50–2.80)	2.10 (1.50–2.80)	2.10 (1.50–2.90)	0.004	0.960
SBP, mmHg	115.52 (15.69)	116.69 (15.68)	118.11 (16.43)	118.79 (16.25)	121.19 (15.82)	< 0.001	< 0.001
DBP, mmHg	68.00 (62.00–75.00)	69.00 (63.00–76.00)	70.00 (64.00–78.00)	71.00 (64.00–79.00)	72.00 (66.00–80.00)	< 0.001	< 0.001
BUN, mmol/l	4.20 (3.48–5.10)	4.20 (3.50–5.10)	4.30 (3.51–5.10)	4.40 (3.60–5.26)	4.40 (3.70–5.30)	< 0.001	< 0.001
CR, μmol/l	73.86 (22.90)	74.98 (26.15)	77.47 (29.89)	78.37 (23.72)	80.46 (22.96)	< 0.001	< 0.001
UA, mmol/l	241.46 (77.19)	251.44 (75.83)	261.97 (79.06)	275.10 (79.84)	288.05 (78.70)	< 0.001	< 0.001
FPG, mmol/l	4.90 (4.65–5.20)	4.93 (4.68–5.21)	4.98 (4.74–5.29)	4.99 (4.73–5.29)	5.06 (4.79–5.35)	< 0.001	< 0.001
TC, mmol/l	4.04 (3.64–4.45)	4.31 (3.89–4.68)	4.38 (3.99–4.73)	4.46 (4.10–4.80)	4.69 (4.38–4.94)	< 0.001	< 0.001
TG, mmol/l	0.76 (0.63–0.96)	0.85 (0.69–1.05)	0.93 (0.74–1.15)	1.01 (0.81–1.26)	1.14 (0.92–1.37)	< 0.001	< 0.001
HDL cholesterol, mmol/l	1.84 (1.66–2.02)	1.66 (1.49–1.80)	1.51 (1.36–1.63)	1.36 (1.25–1.49)	1.21 (1.12–1.31)	< 0.001	< 0.001
LDL cholesterol, mmol/l	1.63 (0.32)	1.97 (0.27)	2.13 (0.27)	2.29 (0.27)	2.55 (0.26)	< 0.001	< 0.001

Values are n(%) or mean (standard deviation) or median (interquartile range)

Abbreviations: *NAFLD* nonalcoholic fatty liver disease, *BMI* body mass index, *BUN* blood urea nitrogen, *Cr* creatinine, *UA* uric acid, *FPG* fasting plasma glucose, *TC* total cholesterol, *TG* triglyceride, *HDL* high-density lipoprotein, *LDL* low-density lipoprotein, *ALP* Alkaline phosphatase, *GGT* gamma-glutamyl transferase, *ALT* alanine aminotransferase, *AST* aspartate aminotransferase, *TP* Total Protein, *ALB* albumin, *GLB* globulin, *TB* Total bilirubin, *DBIL* Direct bilirubin, *DBP* diastolic blood pressure, *SBP* systolic blood pressure

### Association between the LDL/HDL cholesterol ratio and new-onset NAFLD

**Fig. 1:**
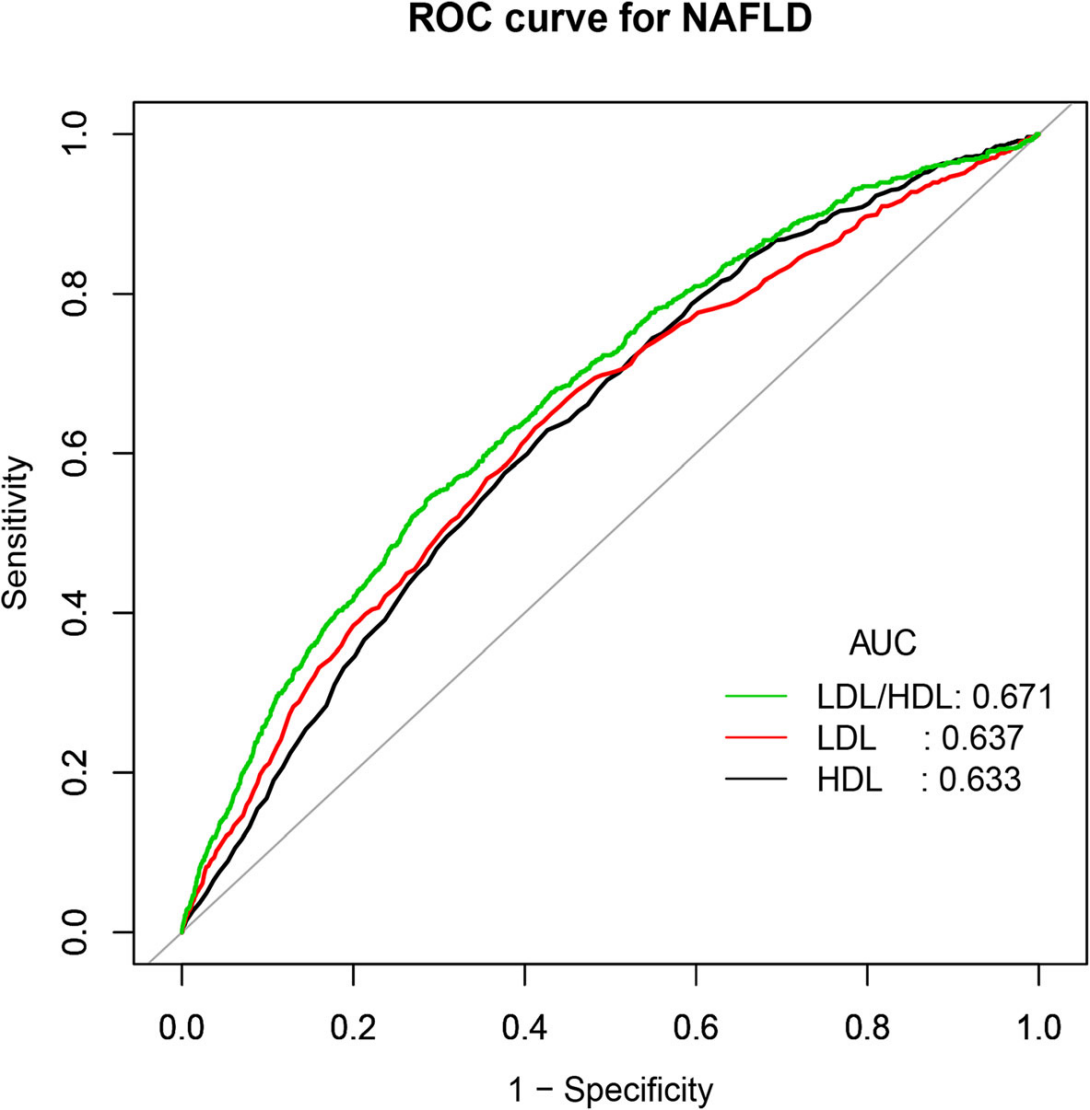
Receiver operating characteristic curve (ROC) of 5-year NAFLD events in non-obese population. AUC: area under the curve; LDL: low density lipoprotein; HDL: high density lipoprotein

Table 2 summarizes the association of the LDL/HDL cholesterol ratio as a categorical variable and continuous variable with new-onset NAFLD. In the five models, the core direction of the relation between the ratio of LDL/HDL cholesterol and NAFLD did not change significantly. In adjustment model IV, for every 1 unit increase in the ratio of LDL/HDL cholesterol, the NAFLD risk increased by 66% (HR: 1.66, 95% CI: 1.38–1.99, *P* trend< 0.001). In the subsidiary analysis, the same steps were used to analyze HDL cholesterol and LDL cholesterol. The results show a positive correlation between LDL cholesterol and new-onset NAFLD, while HDL cholesterol was negatively correlated with new-onset NAFLD risk. In the quintile grouping, it can be observed that the NAFLD risk increases linearly with the increase in LDL cholesterol (*P* trend< 0.001), while with the gradual increase in HDL cholesterol, the NAFLD risk shows a linear downward trend (*P* trend< 0.001) (Additional file 1: Supplementary Tables 3, 4 and 5). In addition, researchers have also drawn an ROC curve to measure the ability of HDL cholesterol, LDL cholesterol and the LDL/HDL cholesterol ratio to predict the risk of new-onset NAFLD (Fig. 1). The areas under the curve of each lipoprotein were as follows: HDL cholesterol: 0.6335 < LDL cholesterol: 0.6367 < LDL/HDL cholesterol ratio: 0.6713 (Table 3). The predictive ability of the ratio of LDL/HDL cholesterol to new-onset NAFLD was better than that of other lipoproteins.

**Table 2:** Hazard ratios for NAFLD events by quintiles of serum lipoproteins and LDL/HDL cholesterol ratio

	HR (95%CI)						
	Multivariable Analysis	Q1	Q2	Q3	Q4	Q5	*P*-trend
HDL cholesterol (mmol/l)							
Crude Model	0.25 (0.19, 0.33)	Ref	0.92 (0.77, 1.10)	0.71 (0.58, 0.86)	0.51 (0.41, 0.63)	0.32 (0.25, 0.41)	< 0.001
Model I	0.26 (0.20, 0.33)	Ref	0.92 (0.77, 1.10)	0.71 (0.58, 0.87)	0.51 (0.41, 0.64)	0.32 (0.25, 0.41)	< 0.001
Model II	0.59 (0.45, 0.77)	Ref	1.05 (0.88, 1.26)	0.95 (0.78, 1.17)	0.85 (0.68, 1.06)	0.60 (0.46, 0.79)	< 0.001
Model III	0.59 (0.44, 0.78)	Ref	1.04 (0.86, 1.25)	0.91 (0.74, 1.11)	0.83 (0.66, 1.04)	0.61 (0.47, 0.80)	< 0.001
Model IV	0.57 (0.42, 0.75)	Ref	0.98 (0.82, 1.18)	0.84 (0.69, 1.04)	0.80 (0.64, 1.01)	0.61 (0.46, 0.79)	< 0.001
LDL cholesterol (mmol/l)							
Crude Model	3.24 (2.70, 3.88)	Ref	1.12 (0.85, 1.49)	1.45 (1.12, 1.89)	1.97 (1.53, 2.52)	3.37 (2.67, 4.26)	< 0.001
Model I	3.21 (2.68, 3.85)	Ref	1.12 (0.85, 1.49)	1.45 (1.11, 1.88)	1.96 (1.53, 2.51)	3.34 (2.64, 4.22)	< 0.001
Model II	3.18 (2.40, 4.21)	Ref	1.26 (0.93, 1.69)	1.48 (1.09, 2.02)	2.00 (1.45, 2.76)	3.10 (2.17, 4.41)	< 0.001
Model III	2.72 (2.03, 3.63)	Ref	1.25 (0.92, 1.70)	1.40 (1.02, 1.92)	1.80 (1.29, 2.50)	2.65 (1.84, 3.82)	< 0.001
Model IV	2.60 (1.95, 3.48)	Ref	1.22 (0.90, 3.48)	1.37 (0.99, 1.88)	1.70 (1.22, 2.37)	2.50 (1.74, 3.60)	< 0.001
LDL/HDL cholesterol ratio							
Crude Model	3.42 (2.93, 3.99)	Ref	1.92 (1.40, 2.63)	2.47 (1.82, 3.35)	3.05 (2.27, 4.10)	5.35 (4.05, 7.07)	< 0.001
Model I	3.38 (2.90, 3.95)	Ref	1.92 (1.40, 2.64)	2.47 (1.82, 3.35)	3.05 (2.27, 4.10)	5.32 (4.02, 7.03)	< 0.001
Model II	1.72 (1.44, 2.06)	Ref	1.63 (1.18, 2.24)	1.89 (1.39, 2.58)	1.87 (1.38, 2.53)	2.43 (1.80, 3.28)	< 0.001
Model III	1.64 (1.37, 1.97)	Ref	1.70 (1.23, 2.35)	1.83 (1.34, 2.51)	1.89 (1.39, 2.56)	2.41 (1.77, 3.27)	< 0.001
Model IV	1.66 (1.38, 1.99)	Ref	1.68 (1.21, 2.33)	1.78 (1.30, 2.44)	1.86 (1.36, 2.55)	2.40 (1.76, 3.27)	< 0.001

Crude model adjusted for none; Model I adjusted for gender and age; Model II adjusted for TC, TG and BMI; Model III adjusted for gender, age, ALP, GGT, ALT, AST, ALB, GLB, DBIL, CR, UA, FPG, TC, TG, height, BMI, SBP and DBP; Model IV adjusted for Model III and TP, TB, BUN

Abbreviations: *NAFLD* nonalcoholic fatty liver disease, *CI* confidence interval, *HR* hazard ratios; other abbreviations as in Table 1

**Table 3:** AUC with the 95% CI of LDL cholesterol, HDL cholesterol and LDL/HDL cholesterol ratio for predicting NAFLD

Variables	AUC	95% CI lower bound	95% CI upper bound	Best threshold	Specificity	Sensitivity
HDL cholesterol	0.6335	0.6147	0.6522	1.4350	0.5734	0.6290
LDL cholesterol	0.6367	0.6168	0.6566	2.2050	0.5879	0.6314
LDL/HDL cholesterol ratio	0.6713	0.6523	0.6904	1.6623	0.7143	0.5422

Abbreviations: *AUC* area under the curve, *CI* confidence interval, *HDL* high-density lipoprotein, *LDL* low-density lipoprotein, *NAFLD* nonalcoholic fatty liver disease

**Fig. 2:**
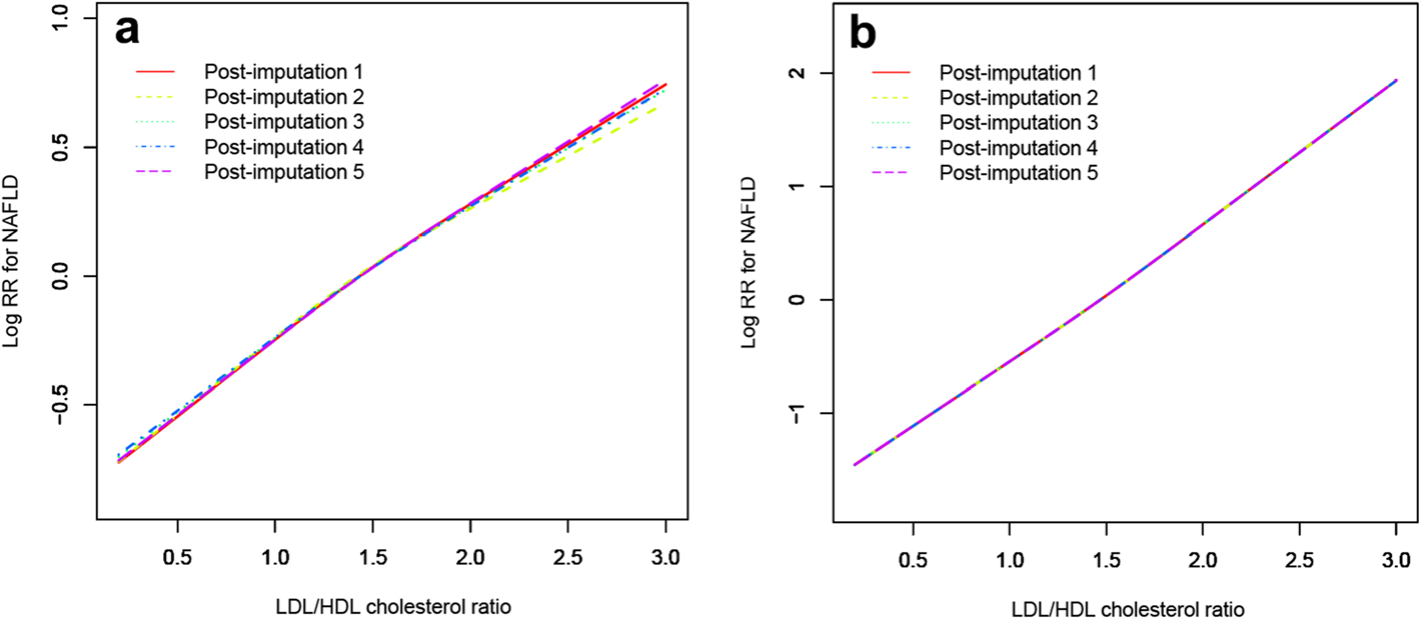
Association between the LDL/HDL cholesterol ratio and the risk of new-onset NAFLD in the unadjusted model (**a**) and adjusted model (**b**). Model adjusted for gender, age, ALP, GGT, ALT, AST, ALB, GLB, DBIL, CR, UA, FPG, TC, TG, height, BMI, SBP and DBP. Different line patterns indicated different data sources

To verify the linear trend between the ratio of LDL/HDL cholesterol and new-onset NAFLD, the researchers established GAM to fit the association between the ratio of LDL/HDL cholesterol as a continuous variable and NAFLD by the cubic spline smoothing technique (Fig. 2). It is obvious from the figure that the linear positive correlation between the ratio of LDL/HDL cholesterol and new-onset NAFLD was stable before and after adjustment.

### Hierarchical analysis and test for interaction

Table 4 evaluates the interaction between the ratio of LDL/HDL cholesterol and age, gender, and glucose metabolism and performs a stratified analysis. Considering the research effect of stratification analysis, stratification factors FPG and age in this study were stratified according to the clinical cutoff point. The significant interaction between the LDL/HDL cholesterol ratio and age was related to NAFLD risk (*P* interaction< 0.05). In the case of a high LDL/HDL cholesterol ratio, the NAFLD risk decreased with increasing age. Additionally, the LDL/HDL cholesterol ratio and NAFLD risk were linearly positively associated in people < 60 years old, while the ratio of LDL/HDL cholesterol and NAFLD risk seemed to be an inverted U-shaped curve in people ≥60 years old (Additional file 2: Supplementary Fig. 1). However, no significant interaction was observed in gender and FPG stratification (*P* interaction> 0.05) (Supplementary Table 6, Additional file 1).

**Table 4:** Stratified associations between LDL/HDL cholesterol ratio and NAFLD by gender, age, and FPG

		LDL/HDL cholesterol ratio (Quintile), HR (95%CI)					
	No. of cases	Q1	Q2	Q3	Q4	Q5	*P*-interaction
Gender							> 0.05
Female	393 (46.73%)	Ref	1.84 (1.13, 3.01)	2.17 (1.36, 3.47)	2.17 (1.36, 3.45)	2.58 (1.64, 4.05)	
Male	448 (53.27%)	1.08 (0.64, 1.85)	1.73 (1.07, 2.82)	1.72 (1.07, 2.77)	1.84 (1.16, 2.91)	2.48 (1.59, 3.88)	
Age, years							< 0.05
≥ 60	127 (15.10%)	Ref	3.31 (1.66, 6.62)	1.57 (0.73, 3.39)	1.42 (0.67, 3.01)	2.23 (1.14, 3.34)	
≥ 45, < 60	247 (29.37%)	1.74 (0.78, 3.87)	2.05 (0.93, 4.48)	2.26 (1.07, 4.75)	2.46 (1.17, 5.16)	3.50 (1.70, 7.20)	
≥ 30, < 45	325 (38.64%)	1.06 (0.41, 2.72)	2.26 (0.94, 5.42)	3.01 (1.27, 7.08)	3.10 (1.33, 7.20)	3.30 (1.43, 7.59)	
< 30	142 (16.88%)	1.78 (0.57, 5.56)	1.67 (0.58, 4.80)	2.66 (0.94, 7.54)	2.81 (1.04, 7.56)	3.96 (1.50, 10.46)	
FPG, mmol/l							> 0.05
≤ 6.1	745 (88.59%)	Ref	1.70 (1.20, 2.41)	1.97 (1.40, 2.76)	1.93 (1.38, 2.70)	2.50 (1.80, 3.47)	
> 6.1	96 (11.41%)	2.08 (1.04, 4.19)	4.16 (2.20, 7.89)	3.24 (1.86, 5.64)	3.74 (2.18, 6.39)	3.96 (2.48, 6.32)	

Adjusted for TC, TG and BMI; Model III adjusted for gender, age, ALP, GGT, ALT, AST, ALB, GLB, DBIL, CR, UA, FPG, TC, TG, height, BMI, SBP and DBP

Note: the model was not adjusted for the stratification variable

Abbreviations: *CI* confidence interval, *HR* hazard ratios, *FPG* fasting plasma glucose, *NAFLD* nonalcoholic fatty liver disease

## Discussion

This study reports the first longitudinal cohort study of the association between the ratio of LDL/HDL cholesterol and new-onset NAFLD risk. During a 5-year prospective follow-up, the cumulative incidence of NAFLD was 25.14%. The study found that in Chinese non-obese people with normal lipids, the LDL/HDL cholesterol ratio was significantly associated with new-onset NAFLD events, which was independent of other risk factors.

Much evidence has shown that HDL cholesterol and LDL cholesterol are closely related to NAFLD [1, 18]. This association was also verified by different models in this study, and the results were the same as the previous conclusion. These conventional lipoproteins have been widely used in the clinic to assess the risk of disease and to judge prognosis. In recent years, many studies have found that the combined indexes of blood lipids, such as the LDL/HDL cholesterol ratio, TG/HDL-cholesterol ratio, and TC/HDL-cholesterol ratio, have better predictive value than single lipoprotein in cardiocerebrovascular diseases, diabetes, and many metabolic diseases [19–21, 31, 32]. However, no studies have been reported on the association between the ratio of LDL/HDL cholesterol and NAFLD, so it is difficult to compare the findings of this study through previous studies. This study was the first to report that there was a positive correlation between the ratio of LDL/HDL cholesterol and new-onset NAFLD risk. This association was linear; even after adjusting for all noncollinear variables, this association remained unchanged. The ROC analysis further evaluated the predictive value of HDL cholesterol, LDL cholesterol and the LDL/HDL cholesterol ratio for new-onset NAFLD. The results showed that the LDL/HDL cholesterol ratio was better than LDL cholesterol and HDL cholesterol in predicting new-onset NAFLD.

This study confirms that a high LDL/HDL cholesterol ratio significantly increases NAFLD risk. Therefore, it may bring beneficial results by effectively reducing the ratio of LDL/HDL cholesterol. A recent nested case-control study of more than 10 million people from South Korea showed that statins could effectively reduce the risk of NAFLD by improving blood lipid metabolism [33]. In other studies of NAFLD treatment, it has also been observed that NAFLD patients benefit from statin treatment [34, 35]. Therefore, the research team suggests that for people with a high LDL/HDL cholesterol ratio, low-dose statins can be used for primary prevention under the guidance of physicians.

This study also investigated the relationship between the ratio of LDL/HDL cholesterol and NAFLD in other subgroups. Studies have shown that there was an interaction between age and LDL/HDL cholesterol ratio-related NAFLD risk. In the group with a higher LDL/HDL cholesterol ratio, the risk of NAFLD decreases with age, which may be related to the unhealthy lifestyle of young people [36]. In the population ≥ 60 years old, the association between them seems to be an inverted U-shaped curve.

This study demonstrates that a high LDL/HDL cholesterol ratio was an independent risk factor for new-onset NAFLD. However, the underlying mechanism is still unclear. In a previous study, Kawamoto et al. found that in non-obese people, the LDL/HDL cholesterol ratio was the best substitute index of insulin resistance (IR) compared to HDL cholesterol, non-HDL cholesterol, TG, LDL cholesterol and the TG/HDL cholesterol ratio [37]. Therefore, researchers speculate that IR may be a potential mediator of NAFLD risk caused by a high LDL/HDL cholesterol ratio. It is well known that the incidence of NAFLD is mainly related to IR and lipid metabolism disorders [1, 2]. In this study, people with abnormal lipid metabolism were excluded. Therefore, a high LDL/HDL cholesterol ratio can be mainly considered a high LDL cholesterol level or low HDL cholesterol level within the normal reference range. In a recent study, Ampuero’s team found that the levels of oxidized LDL cholesterol antibodies/HDL cholesterol were significantly higher in lean NAFLD patients [38]. When the body has elevated levels of oxidized LDL cholesterol antibodies, oxidized LDL cholesterol reduces insulin sensitivity by inhibiting signaling kinase and/or activating the nuclear factor-κB subunit 1 complex responsible for the cellular response to insulin, resulting in IR [39, 40]. High oxidized LDL cholesterol levels will also increase the secretion of the proinflammatory adipocytokines TNF-α and IL6 [40], and the inflammatory response will further lead to IR, which will lead to lipid accumulation and form a vicious cycle [41]. In addition, previous studies have shown that in hepatic steatosis, an alteration in the activity of the transcription factors SREBP-1c and PPAR-alpha is observed. These changes will directly affect the hepatic synthesis of fatty acids, cholesterol and lipoproteins and cause inflammation and antioxidation in the liver [42]. On the other hand, when the HDL cholesterol level is low, the body’s ability to reverse transport cholesterol will decrease, so that the increase in LDL cholesterol level is more prone to LDL cholesterol oxidation [43].

### Study strengths and limitations

The advantage of this study lies in adopting a longitudinal cohort design, large sample size and relatively strict adjustment of statistical variables, which can explain the causal association between the ratio of LDL/HDL cholesterol and new-onset NAFLD. This finding provides a new monitoring tool for preventing and intervening in non-obese healthy people and non-obese NAFLD patients in China.

Although this study has some unique advantages, some limitations are still worth discussing. First, the related indicators of IR were not measured in this study, so it was impossible to evaluate the association between the ratio of LDL/HDL cholesterol and IR. Although it has been found by reviewing the literature that IR may be the intermediate factor of the high LDL/HDL cholesterol ratio causing NAFLD, the complex association still needs to be explored in further studies. Second, although the possible risk factors were widely adjusted in this study, there were still some risk factors that could not be measured or obtained, which may lead to unavoidable residual confusion. Third, previous studies have shown that in non-obese patients, the correlation between metabolic syndrome and NAFLD is significantly stronger than that of obese people [16], which may be the main reason why non-obese people are susceptible to NAFLD. However, due to the particularity of the subjects in this study, metabolic syndrome cannot be diagnosed in the subjects according to the current diagnostic criteria of metabolic syndrome [44], so the correlation cannot be further verified. Finally, the definition of non-obesity in this study was BMI < 25 kg/m^2^, but there were differences in the definition of non-obesity among different ethnic groups [45]; therefore, the results of this study are only for reference for other ethnic groups.

## Conclusion

The LDL/HDL cholesterol ratio is an independent predictor of NAFLD in Chinese non-obese people with normal lipids, and its predictive value is higher than that of other lipoproteins. The best cutoff value of the LDL/HDL cholesterol ratio for predicting NAFLD provided in this study was 1.6623. This longitudinal cohort study’s findings provide an objective and simple marker for clinical workers to assess the risk of NAFLD, which helps identify high-risk groups of NAFLD early for early active intervention.

## Supplementary Information

**Additional file 1: Table S1.** The description of missing data. **Table S2.** Collinearity diagnostics steps. **Table S3.** Results of multivariate linear regression among original data and post-imputation data**. Table S4.** Results of multivariate linear regression among original data and post-imputation data. **Table S5.** Results of multivariate linear regression among original data and post-imputation data. **Table S6.** Stratified associations between LDL/HDL cholesterol ratio and NAFLD by sex, age, and FPG.

**Additional file 2: Figure S1.** Association between the LDL/HDL cholesterol ratio and the risk of new-onset NAFLD in people ≥60 years old. Adjusted for gender, ALP, GGT, ALT, AST, ALB, GLB, DBIL, CR, UA, FPG, TC, TG, height, BMI, SBP and DBP. Different line patterns indicated different data sources.

## Data Availability

Datasets that support the conclusions of this article were available in the [DRYAD] repository. [https://datadryad.org].

## Abbreviations

TC: Total cholesterol
NAFLD: Nonalcoholic fatty liver disease
AST: Aspartate aminotransferase
TG: Triglycerides
BMI: Body mass index
LDL/HDL: Low density lipoprotein to high density lipoprotein
UA: Uric acid
HR: Hazard ratios
S/DBP: Systolic/diastolic blood pressure
DBIL: Direct bilirubin
BUN: Blood urea nitrogen
ALP: Alkaline phosphatase
CI: Confidence interval
FPG: Fasting plasma glucose
TP: Total Protein
ALB: Albumin
ROC: Receiver operating characteristic curve
GLB: Globulin
TB: Total bilirubin
GGT: Gamma glutamyl transferase
ALT: Alanine aminotransferase
GAM: Generalized additive model
IR: Insulin resistance; Cr: creatinine
