# Supplementary material for: LDL/HDL cholesterol ratio is associated with new-onset NAFLD in Chinese non-obese people with normal lipids: a 5-year longitudinal cohort study
Source: Lipids Health Dis. 2021 Mar 25;20:28. doi: 10.1186/s12944-021-01457-1 (PMC7993485; doi:10.1186/s12944-021-01457-1)
Supplement: Supplementary file 1 — Additional file 1: Table S1. The description of missing data. Table S2. Collinearity diagnostics steps. Table S3. Results of multivariate linear regression among original data and post-imputation data. Table S4. Results of multivariate linear regression among original data and post-imputation data. Table S5. Results of multivariate linear regression among original data and post-imputation data. Table S6. Stratified associations between LDL/HDL cholesterol ratio and NAFLD by sex, age, and FPG. [file 12944_2021_1457_MOESM1_ESM.docx]

Supplement Table 1: The description of missing data.

| Variables | Non-missing | Missing |
| --- | --- | --- |
| Gender | 9767 | 0 |
| Age | 9767 | 0 |
| NAFLD | 9767 | 0 |
| ALP | 7224 | 2543 |
| GGT | 7222 | 2545 |
| ALT | 7224 | 2543 |
| AST | 7224 | 2543 |
| TP | 8896 | 871 |
| ALB | 8896 | 871 |
| GLB | 8896 | 871 |
| TB | 6301 | 3466 |
| DBIL | 5389 | 4378 |
| BUN | 9767 | 0 |
| CR | 9767 | 0 |
| UA | 9767 | 0 |
| FPG | 9767 | 0 |
| TC | 9767 | 0 |
| TG | 9767 | 0 |
| HDL cholesterol | 9767 | 0 |
| LDL cholesterol | 9767 | 0 |
| LDL/HDL cholesterol ratio | 9767 | 0 |
| Weight | 9767 | 0 |
| Height | 9767 | 0 |
| BMI | 9767 | 0 |
| SBP | 9759 | 8 |
| DBP | 9759 | 8 |

Supplementary Table 2: Collinearity diagnostics steps.

|  | Step 1 | Step 2 | Step 3 | Step 4 | Step 5 |
| --- | --- | --- | --- | --- | --- |
| LDL/HDL cholesterol ratio | 24.3 | 24.3 | 24.2 | 8.4 | 1.5 |
| ALP | 1.3 | 1.3 | 1.3 | 1.2 | 1.2 |
| GGT | 1.3 | 1.3 | 1.3 | 1.3 | 1.3 |
| ALT | 3.1 | 3.1 | 3.1 | 3.1 | 3.1 |
| AST | 3.2 | 3.2 | 3.2 | 3.2 | 3.2 |
| TP | Inf | NA | NA | NA | NA |
| ALB | Inf | 1.2 | 1.2 | 1.2 | 1.2 |
| GLB | Inf | 1.1 | 1.1 | 1.1 | 1.1 |
| TB | 2.3 | 2.3 | 2.3 | 2.3 | 2.3 |
| DBIL | 2.4 | 2.4 | 2.4 | 2.4 | 2.4 |
| SBP | 2.5 | 2.5 | 2.5 | 2.5 | 2.5 |
| DBP | 2.1 | 2.1 | 2.1 | 2.1 | 2.1 |
| Gender | 1.1 | 1.1 | 1.1 | 1.1 | 1.1 |
| Age | 1.1 | 1.1 | 1.1 | 1.1 | 1.1 |
| BUN | 1.7 | 1.7 | 1.7 | 1.7 | 1.7 |
| CR | 1.7 | 1.7 | 1.7 | 1.7 | 1.6 |
| UA | 1.6 | 1.6 | 1.6 | 1.6 | 1.6 |
| FPG | 1.2 | 1.2 | 1.2 | 1.2 | 1.2 |
| TC | 5.5 | 5.5 | 5.5 | 4.3 | 1.3 |
| TG | 1.6 | 1.6 | 1.6 | 1.5 | 1.4 |
| HDL cholesterol | 10.8 | 10.8 | 10.8 | 7.9 | NA |
| LDL cholesterol | 14.8 | 14.8 | 14.8 | NA | NA |
| Weight | 233.8 | 233.8 | NA | NA | NA |
| Height | 97.7 | 97.7 | 1.4 | 1.4 | 1.4 |
| BMI | 106.9 | 106.9 | 1.3 | 1.3 | 1.3 |

VIF = 1/(1-R^2^).

Supplementary Table 3: Results of multivariate linear regression among original data and post-imputation data.

|  | Original data  HR (95%CI) | Post-imputation 1  HR (95%CI) | Post-imputation 2  HR (95%CI) | Post-imputation 3  HR (95%CI) | Post-imputation 4  HR (95%CI) | Post-imputation 5  HR (95%CI) |
| --- | --- | --- | --- | --- | --- | --- |
| Crude Model |  |  |  |  |  |  |
| LDL/HDL cholesterol ratio | 3.42 (2.93, 3.99) | 3.42 (2.93, 3.99) | 3.42 (2.93, 3.99) | 3.42 (2.93, 3.99) | 3.42 (2.93, 3.99) | 3.42 (2.93, 3.99) |
| LDL/HDL cholesterol ratio (Quintile) | | | | | | |
| Q1 | Ref | Ref | Ref | Ref | Ref | Ref |
| Q2 | 1.92 (1.40, 2.63) | 1.92 (1.40, 2.63) | 1.92 (1.40, 2.63) | 1.92 (1.40, 2.63) | 1.92 (1.40, 2.63) | 1.92 (1.40, 2.63) |
| Q3 | 2.47 (1.82, 3.35) | 2.47 (1.82, 3.35) | 2.47 (1.82, 3.35) | 2.47 (1.82, 3.35) | 2.47 (1.82, 3.35) | 2.47 (1.82, 3.35) |
| Q4 | 3.05 (2.27, 4.10) | 3.05 (2.27, 4.10) | 3.05 (2.27, 4.10) | 3.05 (2.27, 4.10) | 3.05 (2.27, 4.10) | 3.05 (2.27, 4.10) |
| Q5 | 5.35 (4.05, 7.07) | 5.35 (4.05, 7.07) | 5.35 (4.05, 7.07) | 5.35 (4.05, 7.07) | 5.35 (4.05, 7.07) | 5.35 (4.05, 7.07) |
| *P* for trend | <0.0001 | <0.0001 | <0.0001 | <0.0001 | <0.0001 | <0.0001 |
| Model I |  |  |  |  |  |  |
| LDL/HDL cholesterol ratio | 3.38 (2.90, 3.95) | 3.38 (2.90, 3.95) | 3.38 (2.90, 3.95) | 3.38 (2.90, 3.95) | 3.38 (2.90, 3.95) | 3.38 (2.90, 3.95) |
| LDL/HDL cholesterol ratio (Quintile) | | | | | | |
| Q1 | Ref | Ref | Ref | Ref | Ref | Ref |
| Q2 | 1.92 (1.40, 2.64) | 1.92 (1.40, 2.64) | 1.92 (1.40, 2.64) | 1.92 (1.40, 2.64) | 1.92 (1.40, 2.64) | 1.92 (1.40, 2.64) |
| Q3 | 2.47 (1.82, 3.35) | 2.47 (1.82, 3.35) | 2.47 (1.82, 3.35) | 2.47 (1.82, 3.35) | 2.47 (1.82, 3.35) | 2.47 (1.82, 3.35) |
| Q4 | 3.05 (2.27, 4.10) | 3.05 (2.27, 4.10) | 3.05 (2.27, 4.10) | 3.05 (2.27, 4.10) | 3.05 (2.27, 4.10) | 3.05 (2.27, 4.10) |
| Q5 | 5.32 (4.02, 7.03) | 5.32 (4.02, 7.03) | 5.32 (4.02, 7.03) | 5.32 (4.02, 7.03) | 5.32 (4.02, 7.03) | 5.32 (4.02, 7.03) |
| *P* for trend | <0.0001 | <0.0001 | <0.0001 | <0.0001 | <0.0001 | <0.0001 |
| Model II |  |  |  |  |  |  |
| LDL/HDL cholesterol ratio | 1.72 (1.44, 2.06) | 1.72 (1.44, 2.06) | 1.72 (1.44, 2.06) | 1.72 (1.44, 2.06) | 1.72 (1.44, 2.06) | 1.72 (1.44, 2.06) |
| LDL/HDL cholesterol ratio (Quintile) | | | | | | |
| Q1 | Ref | Ref | Ref | Ref | Ref | Ref |
| Q2 | 1.63 (1.18, 2.24) | 1.63 (1.18, 2.24) | 1.63 (1.18, 2.24) | 1.63 (1.18, 2.24) | 1.63 (1.18, 2.24) | 1.63 (1.18, 2.24) |
| Q3 | 1.89 (1.39, 2.58) | 1.89 (1.39, 2.58) | 1.89 (1.39, 2.58) | 1.89 (1.39, 2.58) | 1.89 (1.39, 2.58) | 1.89 (1.39, 2.58) |
| Q4 | 1.87 (1.38, 2.53) | 1.87 (1.38, 2.53) | 1.87 (1.38, 2.53) | 1.87 (1.38, 2.53) | 1.87 (1.38, 2.53) | 1.87 (1.38, 2.53) |
| Q5 | 2.43 (1.80, 3.28) | 2.43 (1.80, 3.28) | 2.43 (1.80, 3.28) | 2.43 (1.80, 3.28) | 2.43 (1.80, 3.28) | 2.43 (1.80, 3.28) |
| *P* for trend | <0.0001 | <0.0001 | <0.0001 | <0.0001 | <0.0001 | <0.0001 |
| Model III |  |  |  |  |  |  |
| LDL/HDL cholesterol ratio | 2.17 (1.69, 2.79) | 1.66 (1.39, 1.99) | 1.60 (1.34, 1.92) | 1.64 (1.37, 1.96) | 1.64 (1.37, 1.96) | 1.67 (1.40, 2.00) |
| LDL/HDL cholesterol ratio (Quintile) | | | | | | |
| Q1 | Ref | Ref | Ref | Ref | Ref | Ref |
| Q2 | 1.63 (1.07, 2.48) | 1.74 (1.26, 2.39) | 1.72 (1.25, 2.38) | 1.71 (1.24, 2.35) | 1.67 (1.21, 2.30) | 1.64 (1.19, 2.27) |
| Q3 | 1.71 (1.12, 2.62) | 1.86 (1.36, 2.54) | 1.83 (1.34, 2.50) | 1.82 (1.33, 2.49) | 1.81 (1.32, 2.47) | 1.84 (1.34, 2.52) |
| Q4 | 2.01 (1.34, 3.01) | 1.91 (1.41, 2.60) | 1.90 (1.40, 2.58) | 1.86 (1.37, 2.53) | 1.86 (1.37, 2.52) | 1.90 (1.40, 2.58) |
| Q5 | 2.97 (1.97, 4.47) | 2.45 (1.80, 3.32) | 2.38 (1.76, 3.24) | 2.41 (1.78, 3.27) | 2.37 (1.74, 3.21) | 2.41 (1.78, 3.28) |
| *P* for trend | <0.0001 | <0.0001 | <0.0001 | <0.0001 | <0.0001 | <0.0001 |
| Model IV |  |  |  |  |  |  |
| LDL/HDL cholesterol ratio | 2.14 (1.66, 2.76) | 1.66 (1.38, 1.99) | 1.63 (1.36, 1.96) | 1.67 (1.39, 2.00) | 1.66 (1.39, 2.00) | 1.67 (1.40, 2.00) |
| LDL/HDL cholesterol ratio (Quintile) | | | | | | |
| Q1 | Ref | Ref | Ref | Ref | Ref | Ref |
| Q2 | 1.62 (1.06, 2.46) | 1.73 (1.26, 2.39) | 1.73 (1.25, 2.39) | 1.67 (1.21, 2.31) | 1.65 (1.19, 2.27) | 1.61 (1.17, 2.22) |
| Q3 | 1.70 (1.11, 2.61) | 1.82 (1.33, 2.49) | 1.81 (1.32, 2.47) | 1.78 (1.30, 2.43) | 1.72 (1.26, 2.35) | 1.78 (1.30, 2.44) |
| Q4 | 2.02 (1.34, 3.04) | 1.90 (1.39, 2.59) | 1.91 (1.40, 2.60) | 1.79 (1.31, 2.44) | 1.82 (1.34, 2.48) | 1.88 (1.38, 2.56) |
| Q5 | 2.93 (1.94, 4.41) | 2.43 (1.79, 3.31) | 2.41 (1.77, 3.27) | 2.41 (1.77, 3.28) | 2.36 (1.73, 3.21) | 2.39 (1.76, 3.25) |
| *P* for trend | <0.0001 | <0.0001 | <0.0001 | <0.0001 | <0.0001 | <0.0001 |

Abbreviations: CI: confidence interval, HR: hazard ratios; other abbreviations as in Supplementary Table ​2; Crude model adjusted for none; Model I adjusted for gender and age; Model II adjusted for TC, TG and BMI; Model III adjusted for gender, age, ALP, GGT, ALT, AST, ALB, GLB, DBIL, CR, UA, FPG, TC, TG, Height, BMI, SBP and DBP; Model IV adjusted for Model III plus TP, TB, BUN.

Supplementary Table 4: Results of multivariate linear regression among original data and post-imputation data.

|  | Original data  HR (95%CI) | Post-imputation 1  HR (95%CI) | Post-imputation 2  HR (95%CI) | Post-imputation 3  HR (95%CI) | Post-imputation 4  HR (95%CI) | Post-imputation 5  HR (95%CI) |
| --- | --- | --- | --- | --- | --- | --- |
| Crude Model |  |  |  |  |  |  |
| HDL cholesterol | 0.25 (0.19, 0.33) | 0.25 (0.19, 0.33) | 0.25 (0.19, 0.33) | 0.25 (0.19, 0.33) | 0.25 (0.19, 0.33) | 0.25 (0.19, 0.33) |
| HDL cholesterol (Quintile) | | | | | | |
| Q1 | Ref | Ref | Ref | Ref | Ref | Ref |
| Q2 | 0.92 (0.77, 1.10) | 0.92 (0.77, 1.10) | 0.92 (0.77, 1.10) | 0.92 (0.77, 1.10) | 0.92 (0.77, 1.10) | 0.92 (0.77, 1.10) |
| Q3 | 0.71 (0.58, 0.86) | 0.71 (0.58, 0.86) | 0.71 (0.58, 0.86) | 0.71 (0.58, 0.86) | 0.71 (0.58, 0.86) | 0.71 (0.58, 0.86) |
| Q4 | 0.51 (0.41, 0.63) | 0.51 (0.41, 0.63) | 0.51 (0.41, 0.63) | 0.51 (0.41, 0.63) | 0.51 (0.41, 0.63) | 0.51 (0.41, 0.63) |
| Q5 | 0.32 (0.25, 0.41) | 0.32 (0.25, 0.41) | 0.32 (0.25, 0.41) | 0.32 (0.25, 0.41) | 0.32 (0.25, 0.41) | 0.32 (0.25, 0.41) |
| *P* for trend | <0.0001 | <0.0001 | 0.0004 | 0.0002 | 0.0002 | <0.0001 |
| Model I |  |  |  |  |  |  |
| HDL cholesterol | 0.26 (0.20, 0.33) | 0.26 (0.20, 0.33) | 0.26 (0.20, 0.33) | 0.26 (0.20, 0.33) | 0.26 (0.20, 0.33) | 0.26 (0.20, 0.33) |
| HDL cholesterol (Quintile) | | | | | | |
| Q1 | Ref | Ref | Ref | Ref | Ref | Ref |
| Q2 | 0.92 (0.77, 1.10) | 0.92 (0.77, 1.10) | 0.92 (0.77, 1.10) | 0.92 (0.77, 1.10) | 0.92 (0.77, 1.10) | 0.92 (0.77, 1.10) |
| Q3 | 0.71 (0.58, 0.87) | 0.71 (0.58, 0.87) | 0.71 (0.58, 0.87) | 0.71 (0.58, 0.87) | 0.71 (0.58, 0.87) | 0.71 (0.58, 0.87) |
| Q4 | 0.51 (0.41, 0.64) | 0.51 (0.41, 0.64) | 0.51 (0.41, 0.64) | 0.51 (0.41, 0.64) | 0.51 (0.41, 0.64) | 0.51 (0.41, 0.64) |
| Q5 | 0.32 (0.25, 0.41) | 0.32 (0.25, 0.41) | 0.32 (0.25, 0.41) | 0.32 (0.25, 0.41) | 0.32 (0.25, 0.41) | 0.32 (0.25, 0.41) |
| *P* for trend | <0.0001 | <0.0001 | <0.0001 | <0.0001 | <0.0001 | <0.0001 |
| Model II |  |  |  |  |  |  |
| HDL cholesterol | 0.59 (0.45, 0.77) | 0.59 (0.45, 0.77) | 0.59 (0.45, 0.77) | 0.59 (0.45, 0.77) | 0.59 (0.45, 0.77) | 0.59 (0.45, 0.77) |
| HDL cholesterol (Quintile) | | | | | | |
| Q1 | Ref | Ref | Ref | Ref | Ref | Ref |
| Q2 | 1.05 (0.88, 1.26) | 1.05 (0.88, 1.26) | 1.05 (0.88, 1.26) | 1.05 (0.88, 1.26) | 1.05 (0.88, 1.26) | 1.05 (0.88, 1.26) |
| Q3 | 0.95 (0.78, 1.17) | 0.95 (0.78, 1.17) | 0.95 (0.78, 1.17) | 0.95 (0.78, 1.17) | 0.95 (0.78, 1.17) | 0.95 (0.78, 1.17) |
| Q4 | 0.85 (0.68, 1.06) | 0.85 (0.68, 1.06) | 0.85 (0.68, 1.06) | 0.85 (0.68, 1.06) | 0.85 (0.68, 1.06) | 0.85 (0.68, 1.06) |
| Q5 | 0.60 (0.46, 0.79) | 0.60 (0.46, 0.79) | 0.60 (0.46, 0.79) | 0.60 (0.46, 0.79) | 0.60 (0.46, 0.79) | 0.60 (0.46, 0.79) |
| *P* for trend | 0.0002 | 0.0002 | 0.0002 | 0.0002 | 0.0002 | 0.0000 |
| Model III |  |  |  |  |  |  |
| HDL cholesterol | 0.33 (0.21, 0.50) | 0.58 (0.44, 0.77) | 0.60 (0.46, 0.80) | 0.59 (0.45, 0.78) | 0.59 (0.44, 0.77) | 0.57 (0.43, 0.75) |
| HDL cholesterol (Quintile) | | | | | | |
| Q1 | Ref | Ref | Ref | Ref | Ref | Ref |
| Q2 | 0.90 (0.70, 1.16) | 1.02 (0.85, 1.22) | 1.05 (0.87, 1.26) | 1.06 (0.89, 1.27) | 1.04 (0.87, 1.25) | 1.02 (0.85, 1.22) |
| Q3 | 0.63 (0.47, 0.84) | 0.89 (0.72, 1.09) | 0.92 (0.75, 1.13) | 0.92 (0.75, 1.13) | 0.90 (0.74, 1.11) | 0.90 (0.73, 1.10) |
| Q4 | 0.63 (0.46, 0.87) | 0.83 (0.66, 1.04) | 0.84 (0.67, 1.06) | 0.83 (0.66, 1.05) | 0.83 (0.66, 1.04) | 0.81 (0.65, 1.02) |
| Q5 | 0.42 (0.29, 0.61) | 0.60 (0.46, 0.79) | 0.63 (0.48, 0.82) | 0.62 (0.48, 0.82) | 0.61 (0.47, 0.81) | 0.60 (0.46, 0.79) |
| *P* for trend | <0.0001 | 0.0002 | 0.0007 | 0.0004 | 0.0003 | 0.0002 |
| Model IV |  |  |  |  |  |  |
| HDL cholesterol | 0.33 (0.22, 0.51) | 0.57 (0.43, 0.76) | 0.58 (0.43, 0.76) | 0.57 (0.43, 0.75) | 0.56 (0.42, 0.75) | 0.55 (0.41, 0.73) |
| HDL cholesterol (Quintile) | | | | | | |
| Q1 | Ref | Ref | Ref | Ref | Ref | Ref |
| Q2 | 0.85 (0.66, 1.10) | 0.98 (0.82, 1.18) | 0.99 (0.83, 1.19) | 1.01 (0.84, 1.21) | 0.98 (0.82, 1.18) | 0.96 (0.80, 1.15) |
| Q3 | 0.62 (0.46, 0.82) | 0.84 (0.69, 1.04) | 0.86 (0.70, 1.06) | 0.85 (0.70, 1.05) | 0.84 (0.68, 1.03) | 0.85 (0.69, 1.04) |
| Q4 | 0.63 (0.46, 0.86) | 0.82 (0.65, 1.02) | 0.81 (0.64, 1.01) | 0.80 (0.64, 1.01) | 0.80 (0.64, 1.01) | 0.78 (0.62, 0.98) |
| Q5 | 0.43 (0.29, 0.62) | 0.61 (0.46, 0.80) | 0.61 (0.47, 0.80) | 0.61 (0.46, 0.80) | 0.60 (0.46, 0.79) | 0.60 (0.46, 0.78) |
| *P* for trend | <0.0001 | <0.0001 | <0.0001 | <0.0001 | <0.0001 | <0.0001 |

Abbreviations: CI: confidence interval, HR: hazard ratios; other abbreviations as in Supplementary Table ​2; Crude model adjusted for none; Model I adjusted for gender and age; Model II adjusted for TC, TG and BMI; Model III adjusted for gender, age, ALP, GGT, ALT, AST, ALB, GLB, DBIL, CR, UA, FPG, TC, TG, Height, BMI, SBP and DBP; Model IV adjusted for Model III plus TP, TB BUN.

Supplementary Table 5: Results of multivariate linear regression among original data and post-imputation data.

|  | Original data  HR (95%CI) | Post-imputation 1  HR (95%CI) | Post-imputation 2  HR (95%CI) | Post-imputation 3  HR (95%CI) | Post-imputation 4  HR (95%CI) | Post-imputation 5  HR (95%CI) |
| --- | --- | --- | --- | --- | --- | --- |
| Crude Model |  |  |  |  |  |  |
| LDL cholesterol | 3.24 (2.70, 3.88) | 3.24 (2.70, 3.88) | 3.24 (2.70, 3.88) | 3.24 (2.70, 3.88) | 3.24 (2.70, 3.88) | 3.24 (2.70, 3.88) |
| LDL cholesterol (Quintile) | | | | | | |
| Q1 | Ref | Ref | Ref | Ref | Ref | Ref |
| Q2 | 1.12 (0.85, 1.49) | 1.12 (0.85, 1.49) | 1.12 (0.85, 1.49) | 1.12 (0.85, 1.49) | 1.12 (0.85, 1.49) | 1.12 (0.85, 1.49) |
| Q3 | 1.45 (1.12, 1.89) | 1.45 (1.12, 1.89) | 1.45 (1.12, 1.89) | 1.45 (1.12, 1.89) | 1.45 (1.12, 1.89) | 1.45 (1.12, 1.89) |
| Q4 | 1.97 (1.53, 2.52) | 1.97 (1.53, 2.52) | 1.97 (1.53, 2.52) | 1.97 (1.53, 2.52) | 1.97 (1.53, 2.52) | 1.97 (1.53, 2.52) |
| Q5 | 3.37 (2.67, 4.26) | 3.37 (2.67, 4.26) | 3.37 (2.67, 4.26) | 3.37 (2.67, 4.26) | 3.37 (2.67, 4.26) | 3.37 (2.67, 4.26) |
| *P* for trend | <0.0001 | <0.0001 | 0.0004 | 0.0002 | 0.0002 | <0.0001 |
| Model I |  |  |  |  |  |  |
| LDL cholesterol | 3.21 (2.68, 3.85) | 3.21 (2.68, 3.85) | 3.21 (2.68, 3.85) | 3.21 (2.68, 3.85) | 3.21 (2.68, 3.85) | 3.21 (2.68, 3.85) |
| LDL cholesterol (Quintile) | | | | | | |
| Q1 | Ref | Ref | Ref | Ref | Ref | Ref |
| Q2 | 1.12 (0.85, 1.49) | 1.12 (0.85, 1.49) | 1.12 (0.85, 1.49) | 1.12 (0.85, 1.49) | 1.12 (0.85, 1.49) | 1.12 (0.85, 1.49) |
| Q3 | 1.45 (1.11, 1.88) | 1.45 (1.11, 1.88) | 1.45 (1.11, 1.88) | 1.45 (1.11, 1.88) | 1.45 (1.11, 1.88) | 1.45 (1.11, 1.88) |
| Q4 | 1.96 (1.53, 2.51) | 1.96 (1.53, 2.51) | 1.96 (1.53, 2.51) | 1.96 (1.53, 2.51) | 1.96 (1.53, 2.51) | 1.96 (1.53, 2.51) |
| Q5 | 3.34 (2.64, 4.22) | 3.34 (2.64, 4.22) | 3.34 (2.64, 4.22) | 3.34 (2.64, 4.22) | 3.34 (2.64, 4.22) | 3.34 (2.64, 4.22) |
| *P* for trend | <0.0001 | <0.0001 | <0.0001 | <0.0001 | <0.0001 | <0.0001 |
| Model II |  |  |  |  |  |  |
| LDL cholesterol | 3.18 (2.40, 4.21) | 3.18 (2.40, 4.21) | 3.18 (2.40, 4.21) | 3.18 (2.40, 4.21) | 3.18 (2.40, 4.21) | 3.18 (2.40, 4.21) |
| LDL cholesterol (Quintile) | | | | | | |
| Q1 | Ref | Ref | Ref | Ref | Ref | Ref |
| Q2 | 1.26 (0.93, 1.69) | 1.26 (0.93, 1.69) | 1.26 (0.93, 1.69) | 1.26 (0.93, 1.69) | 1.26 (0.93, 1.69) | 1.26 (0.93, 1.69) |
| Q3 | 1.48 (1.09, 2.02) | 1.48 (1.09, 2.02) | 1.48 (1.09, 2.02) | 1.48 (1.09, 2.02) | 1.48 (1.09, 2.02) | 1.48 (1.09, 2.02) |
| Q4 | 2.00 (1.45, 2.76) | 2.00 (1.45, 2.76) | 2.00 (1.45, 2.76) | 2.00 (1.45, 2.76) | 2.00 (1.45, 2.76) | 2.00 (1.45, 2.76) |
| Q5 | 3.10 (2.17, 4.41) | 3.10 (2.17, 4.41) | 3.10 (2.17, 4.41) | 3.10 (2.17, 4.41) | 3.10 (2.17, 4.41) | 3.10 (2.17, 4.41) |
| *P* for trend | <0.0001 | <0.0001 | <0.0001 | <0.0001 | <0.0001 | <0.0001 |
| Model III |  |  |  |  |  |  |
| LDL cholesterol | 3.05 (2.03, 4.56) | 2.77 (2.08, 3.70) | 2.64 (1.98, 3.53) | 2.74 (2.05, 3.65) | 2.69 (2.02, 3.59) | 2.75 (2.07, 3.67) |
| LDL cholesterol (Quintile) | | | | | | |
| Q1 | Ref | Ref | Ref | Ref | Ref | Ref |
| Q2 | 1.12 (0.74, 1.69) | 1.27 (0.93, 1.71) | 1.26 (0.93, 1.71) | 1.25 (0.93, 1.70) | 1.21 (0.90, 1.64) | 1.28 (0.95, 1.73) |
| Q3 | 1.31 (0.85, 2.01) | 1.42 (1.04, 1.95) | 1.42 (1.04, 1.94) | 1.39 (1.02, 1.91) | 1.37 (1.00, 1.88) | 1.38 (1.00, 1.89) |
| Q4 | 1.55 (0.98, 2.44) | 1.76 (1.27, 2.45) | 1.84 (1.32, 2.55) | 1.85 (1.33, 2.56) | 1.75 (1.26, 2.43) | 1.78 (1.29, 2.48) |
| Q5 | 2.62 (1.58, 4.34) | 2.71 (1.88, 3.90) | 2.63 (1.83, 3.78) | 2.65 (1.84, 3.80) | 2.58 (1.79, 3.70) | 2.70 (1.88, 3.88) |
| *P* for trend | <0.0001 | <0.0001 | <0.0001 | <0.0001 | <0.0001 | <0.0001 |
| Model IV |  |  |  |  |  |  |
| LDL cholesterol | 2.96 (1.97, 4.45) | 2.65 (1.98, 3.54) | 2.56 (1.91, 3.42) | 2.63 (1.97, 3.52) | 2.59 (1.94, 3.45) | 2.59 (1.94, 3.45) |
| LDL cholesterol (Quintile) | | | | | | |
| Q1 | Ref | Ref | Ref | Ref | Ref | Ref |
| Q2 | 1.09 (0.72, 1.64) | 1.21 (0.89, 1.64) | 1.21 (0.89, 1.64) | 1.23 (0.91, 1.67) | 1.18 (0.87, 1.60) | 1.26 (0.93, 1.70) |
| Q3 | 1.33 (0.87, 2.05) | 1.38 (1.01, 1.90) | 1.39 (1.01, 1.90) | 1.37 (1.00, 1.88) | 1.34 (0.98, 1.84) | 1.35 (0.98, 1.85) |
| Q4 | 1.43 (0.90, 2.28) | 1.66 (1.19, 2.31) | 1.74 (1.25, 2.41) | 1.75 (1.26, 2.43) | 1.67 (1.20, 2.32) | 1.68 (1.21, 2.34) |
| Q5 | 2.41 (1.45, 4.00) | 2.53 (1.75, 3.65) | 2.46 (1.71, 3.53) | 2.53 (1.76, 3.63) | 2.50 (1.74, 3.59) | 2.49 (1.73, 3.58) |
| *P* for trend | <0.0001 | <0.0001 | <0.0001 | <0.0001 | <0.0001 | <0.0001 |

Abbreviations: CI: confidence interval, HR: hazard ratios; other abbreviations as in Supplementary Table ​2; Crude model adjusted for none; Model I adjusted for gender and age; Model II adjusted for TC, TG and BMI; Model III adjusted for gender, age, ALP, GGT, ALT, AST, ALB, GLB, DBIL, CR, UA, FPG, TC, TG, Height, BMI, SBP and DBP; Model IV adjusted for Model III plus TP, TB BUN.

Supplementary Table 6: Stratified associations between LDL/HDL cholesterol ratio and NAFLD by sex, age, and FPG.

| LDL/HDL cholesterol ratio (Quintile) | | | | | |  |
| --- | --- | --- | --- | --- | --- | --- |
|  | Q1 | Q2 | Q3 | Q4 | Q5 | *P*-interaction |
| Sex |  |  |  |  |  |  |
| Original data |  |  |  |  |  | 0.8261 |
| Female | Ref | 1.27 (0.66, 2.44) | 1.47 (0.79, 2.73) | 1.84 (1.02, 3.33) | 2.77 (1.53, 5.00) |  |
| Male | 0.63 (0.32, 1.23) | 1.21 (0.65, 2.24) | 1.22 (0.64, 2.32) | 1.32 (0.72, 2.42) | 1.94 (1.08, 3.51) |  |
| Post-imputation 1 |  |  |  |  |  | 0.7264 |
| Female | Ref | 1.90 (1.17, 3.08) | 2.24 (1.41, 3.56) | 2.24 (1.42, 3.55) | 2.65 (1.69, 4.15) |  |
| Male | 1.10 (0.65, 1.86) | 1.79 (1.11, 2.89) | 1.74 (1.08, 2.79) | 1.85 (1.17, 2.93) | 2.53 (1.62, 3.95) |  |
| Post-imputation 2 |  |  |  |  |  | 0.6949 |
| Female | Ref | 1.92 (1.18, 3.12) | 2.21 (1.39, 3.52) | 2.26 (1.43, 3.58) | 2.59 (1.65, 4.05) |  |
| Male | 1.13 (0.67, 1.91) | 1.79 (1.11, 2.90) | 1.76 (1.10, 2.82) | 1.87 (1.18, 2.95) | 2.53 (1.62, 3.95) |  |
| Post-imputation 3 |  |  |  |  |  | 0.9250 |
| Female | Ref | 1.86 (1.14, 3.02) | 2.10 (1.32, 3.35) | 2.11 (1.33, 3.34) | 2.63 (1.68, 4.12) |  |
| Male | 1.10 (0.65, 1.86) | 1.76 (1.09, 2.84) | 1.77 (1.11, 2.83) | 1.86 (1.18, 2.93) | 2.48 (1.59, 3.86) |  |
| Post-imputation 4 |  |  |  |  |  | 0.6883 |
| Female | Ref | 1.80 (1.11, 2.93) | 2.20 (1.38, 3.50) | 2.12 (1.34, 3.36) | 2.54 (1.62, 3.98) |  |
| Male | 1.08 (0.64, 1.82) | 1.70 (1.05, 2.75) | 1.64 (1.02, 2.63) | 1.80 (1.14, 2.84) | 2.42 (1.55, 3.78) |  |
| Post-imputation 5 |  |  |  |  |  | 0.8028 |
| Female | Ref | 1.74 (1.07, 2.82) | 2.12 (1.33, 3.38) | 2.11 (1.34, 3.34) | 2.49 (1.59, 3.90) |  |
| Male | 1.04 (0.61, 1.75) | 1.64 (1.01, 2.64) | 1.68 (1.04, 2.69) | 1.81 (1.14, 2.85) | 2.44 (1.57, 3.81) |  |
| Age |  |  |  |  |  |  |
| Original data |  |  |  |  |  | 0.0283 |
| ≥60 | Ref | 3.32 (1.48, 7.44) | 1.29 (0.48, 3.46) | 1.64 (0.66, 4.08) | 2.72 (1.21, 6.09) |  |
| ≥45, <60 | 1.42 (0.53, 3.85) | 1.42 (0.54, 3.75) | 2.35 (0.93, 5.92) | 2.00 (0.79, 5.04) | 3.80 (1.53, 9.44) |  |
| ≥30, <45 | 0.95 (0.28, 3.17) | 1.52 (0.48, 4.80) | 1.99 (0.65, 6.15) | 2.77 (0.95, 8.08) | 3.32 (1.15, 9.60) |  |
| <30 | 1.15 (0.26, 5.03) | 1.22 (0.29, 5.16) | 1.63 (0.40, 6.59) | 2.52 (0.71, 9.02) | 3.80 (1.07, 13.46) |  |
| Post-imputation 1 |  |  |  |  |  | 0.0045 |
| ≥60 | Ref | 3.46 (1.74, 6.88) | 1.65 (0.77, 3.54) | 1.44 (0.68, 3.05) | 2.28 (1.17, 4.44) |  |
| ≥45, <60 | 1.67 (0.75, 3.72) | 2.06 (0.95, 4.49) | 2.22 (1.06, 4.65) | 2.35 (1.13, 4.90) | 3.34 (1.63, 6.82) |  |
| ≥30, <45 | 1.00 (0.39, 2.56) | 2.16 (0.91, 5.15) | 2.86 (1.22, 6.70) | 2.99 (1.29, 6.91) | 3.21 (1.40, 7.35) |  |
| <30 | 1.66 (0.54, 5.16) | 1.56 (0.55, 4.43) | 2.41 (0.86, 6.74) | 2.67 (1.00, 7.13) | 3.69 (1.41, 9.67) |  |
| Post-imputation 2 |  |  |  |  |  | 0.0048 |
| ≥60 | Ref | 3.39 (1.71, 6.74) | 1.51 (0.70, 3.23) | 1.41 (0.67, 2.98) | 2.14 (1.10, 4.16) |  |
| ≥45, <60 | 1.74 (0.78, 3.85) | 2.05 (0.94, 4.47) | 2.23 (1.06, 4.66) | 2.53 (1.21, 5.28) | 3.46 (1.69, 7.08) |  |
| ≥30, <45 | 1.03 (0.40, 2.63) | 2.30 (0.97, 5.50) | 3.00 (1.28, 7.05) | 3.03 (1.31, 7.00) | 3.25 (1.42, 7.47) |  |
| <30 | 1.75 (0.56, 5.45) | 1.62 (0.57, 4.62) | 2.65 (0.95, 7.40) | 2.77 (1.03, 7.43) | 3.79 (1.45, 9.94) |  |
| Post-imputation 3 |  |  |  |  |  | 0.0106 |
| ≥60 | Ref | 3.36 (1.69, 6.69) | 1.59 (0.74, 3.41) | 1.43 (0.68, 3.03) | 2.22 (1.14, 4.33) |  |
| ≥45, <60 | 1.82 (0.82, 4.04) | 2.12 (0.97, 4.65) | 2.41 (1.15, 5.05) | 2.55 (1.22, 5.34) | 3.70 (1.80, 7.59) |  |
| ≥30, <45 | 1.12 (0.44, 2.88) | 2.43 (1.01, 5.81) | 3.10 (1.32, 7.31) | 3.30 (1.42, 7.68) | 3.51 (1.52, 8.08) |  |
| <30 | 1.92 (0.61, 6.00) | 1.83 (0.64, 5.25) | 2.85 (1.01, 8.00) | 2.85 (1.06, 7.68) | 4.28 (1.62, 11.27) |  |
| Post-imputation 4 |  |  |  |  |  | 0.0048 |
| ≥60 | Ref | 3.28 (1.65, 6.52) | 1.51 (0.70, 3.23) | 1.38 (0.65, 2.92) | 2.20 (1.13, 4.29) |  |
| ≥45, <60 | 1.73 (0.78, 3.83) | 2.00 (0.92, 4.37) | 2.18 (1.04, 4.57) | 2.36 (1.13, 4.93) | 3.45 (1.68, 7.07) |  |
| ≥30, <45 | 1.05 (0.41, 2.71) | 2.19 (0.92, 5.23) | 3.01 (1.28, 7.05) | 3.04 (1.31, 7.05) | 3.19 (1.39, 7.31) |  |
| <30 | 1.75 (0.56, 5.45) | 1.64 (0.58, 4.69) | 2.61 (0.93, 7.31) | 2.79 (1.04, 7.49) | 3.86 (1.47, 10.12) |  |
| Post-imputation 5 |  |  |  |  |  | 0.0113 |
| ≥60 | Ref | 3.09 (1.56, 6.15) | 1.61 (0.75, 3.45) | 1.44 (0.68, 3.05) | 2.29 (1.18, 4.46) |  |
| ≥45, <60 | 1.75 (0.79, 3.88) | 2.03 (0.93, 4.43) | 2.25 (1.07, 4.71) | 2.53 (1.21, 5.28) | 3.55 (1.73, 7.26) |  |
| ≥30, <45 | 1.09 (0.43, 2.81) | 2.24 (0.94, 5.35) | 3.08 (1.31, 7.23) | 3.13 (1.35, 7.25) | 3.33 (1.45, 7.62) |  |
| <30 | 1.82 (0.59, 5.67) | 1.72 (0.60, 4.90) | 2.85 (1.02, 7.96) | 2.98 (1.11, 7.97) | 4.19 (1.60, 10.97) |  |
| FPG |  |  |  |  |  |  |
| Original data |  |  |  |  |  | 0.4459 |
| ≤6.1 | Ref | 1.55 (0.97, 2.49) | 1.56 (0.97, 2.53) | 2.00 (1.28, 3.14) | 3.12 (1.99, 4.87) |  |
| >6.1 | 1.82 (0.84, 3.95) | 3.95 (1.91, 8.14) | 4.61 (2.36, 9.00) | 3.70 (1.86, 7.34) | 4.08 (2.27, 7.33) |  |
| Post-imputation 1 |  |  |  |  |  | 0.8694 |
| ≤6.1 | Ref | 1.74 (1.23, 2.47) | 1.98 (1.41, 2.78) | 1.95 (1.40, 2.72) | 2.52 (1.82, 3.49) |  |
| >6.1 | 2.06 (1.03, 4.13) | 4.25 (2.26, 7.99) | 3.53 (2.06, 6.05) | 3.72 (2.19, 6.33) | 4.19 (2.64, 6.66) |  |
| Post-imputation 2 |  |  |  |  |  | 0.6270 |
| ≤6.1 | Ref | 1.72 (1.22, 2.44) | 1.96 (1.40, 2.75) | 1.95 (1.40, 2.72) | 2.49 (1.79, 3.45) |  |
| >6.1 | 2.13 (1.06, 4.26) | 4.48 (2.39, 8.42) | 3.39 (1.98, 5.82) | 3.88 (2.28, 6.60) | 3.82 (2.40, 6.07) |  |
| Post-imputation 3 |  |  |  |  |  | 0.7673 |
| ≤6.1 | Ref | 1.71 (1.21, 2.42) | 1.95 (1.39, 2.74) | 1.91 (1.37, 2.66) | 2.50 (1.80, 3.47) |  |
| >6.1 | 2.09 (1.04, 4.18) | 4.11 (2.18, 7.74) | 3.16 (1.84, 5.42) | 3.81 (2.24, 6.48) | 4.01 (2.52, 6.37) |  |
| Post-imputation 4 |  |  |  |  |  | 0.7971 |
| ≤6.1 | Ref | 1.67 (1.18, 2.36) | 1.94 (1.38, 2.72) | 1.89 (1.36, 2.64) | 2.45 (1.76, 3.40) |  |
| >6.1 | 1.98 (0.99, 3.96) | 3.95 (2.10, 7.43) | 3.01 (1.76, 5.15) | 3.49 (2.05, 5.93) | 3.87 (2.44, 6.14) |  |
| Post-imputation 5 |  |  |  |  |  | 0.6913 |
| ≤6.1 | Ref | 1.67 (1.18, 2.36) | 2.00 (1.43, 2.80) | 1.96 (1.40, 2.72) | 2.52 (1.82, 3.50) |  |
| >6.1 | 2.16 (1.08, 4.33) | 4.05 (2.15, 7.62) | 3.16 (1.84, 5.41) | 3.80 (2.24, 6.46) | 3.91 (2.46, 6.21) |  |

Adjusted for gender, age, ALP, GGT, ALT, AST, ALB, GLB, DBIL, CR, UA, FPG, TC, TG, Height, BMI, SBP and DBP;

Note: the model is not adjusted for the stratification variable.
