# Supplementary figures and images for: LDL/HDL cholesterol ratio is associated with new-onset NAFLD in Chinese non-obese people with normal lipids: a 5-year longitudinal cohort study
Source: Lipids Health Dis. 2021 Mar 25;20:28. doi: 10.1186/s12944-021-01457-1 (PMC7993485; doi:10.1186/s12944-021-01457-1)

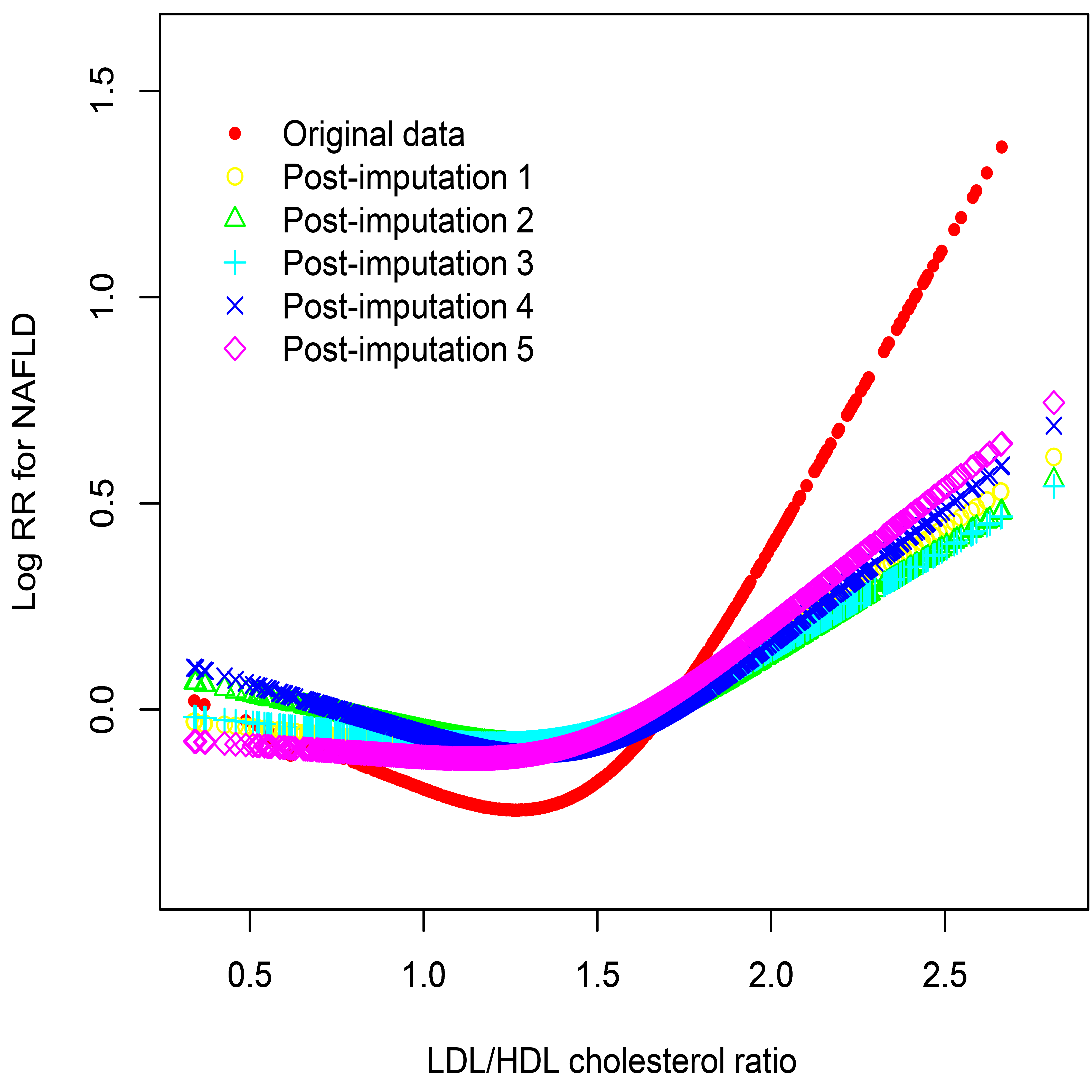

Supplement: Supplementary file 2 — Additional file 2: Figure S1. Association between the LDL/HDL cholesterol ratio and the risk of new-onset NAFLD in people ≥60 years old. Adjusted for gender, ALP, GGT, ALT, AST, ALB, GLB, DBIL, CR, UA, FPG, TC, TG, height, BMI, SBP and DBP. Different line patterns indicated different data sources. [file 12944_2021_1457_MOESM2_ESM.tif]
